# DAILY SLEEP, SOCIAL ENCOUNTERS, AND MOMENTARY LONELINESS IN LATE LIFE

**DOI:** 10.1093/geroni/igae098.1882

**Published:** 2024-12-31

**Authors:** Zexi Zhou, Karen Fingerman

**Affiliations:** The University of Texas Austin, Austin, Texas, United States; The University of Texas Austin, Austin, Texas, United States

## Abstract

Social experiences have implications for well-being in late life, and everyday sleep may play an important role in shaping those experiences. This study examines the associations between sleep, quality of social encounters, and momentary loneliness in older adults’ daily life. We used the ecological momentary assessment (EMA) data over 5 to 6 days from the Daily Experiences and Well-being Study (N = 287, Mage = 74.88). Every morning, older adults reported their sleep the prior night (e.g., duration, subjective quality, disturbances). At each three-hour assessment, they reported encounters with close social partners and partners not identified as close (i.e., less close partners), indicated the pleasantness and stressfulness of each encounter, and rated their feelings of loneliness. Multilevel models showed that older adults who overall reported higher sleep quality or fewer sleep disturbances also had more pleasant encounters and fewer stressful encounters with both close and less close social partners across the study period. More pleasant and less stressful social encounters with close partners were associated with feeling less lonely especially on days when older adults had better sleep the prior night, compared to days when they had worse prior night’s sleep. Higher-quality encounters with less close social partners were associated with feeling less lonely throughout the day regardless of prior night’s sleep. Findings suggest the promotive and protective role that better sleep plays in older adults’ everyday social life, and highlight the robust benefits that positive social encounters with less close ties may confer in mitigating loneliness in late life.

